# Supplementary material for: A Single-Cell Transcriptome Atlas of Epithelial Subpopulations in HPV-Positive and HPV-Negative Head and Neck Cancers
Source: Viruses. 2025 Mar 24;17(4):461. doi: 10.3390/v17040461 (PMC12031417; doi:10.3390/v17040461)
Supplement: Supplementary file 1 [file viruses-17-00461-s001.zip › Supplemental Figures.pdf]

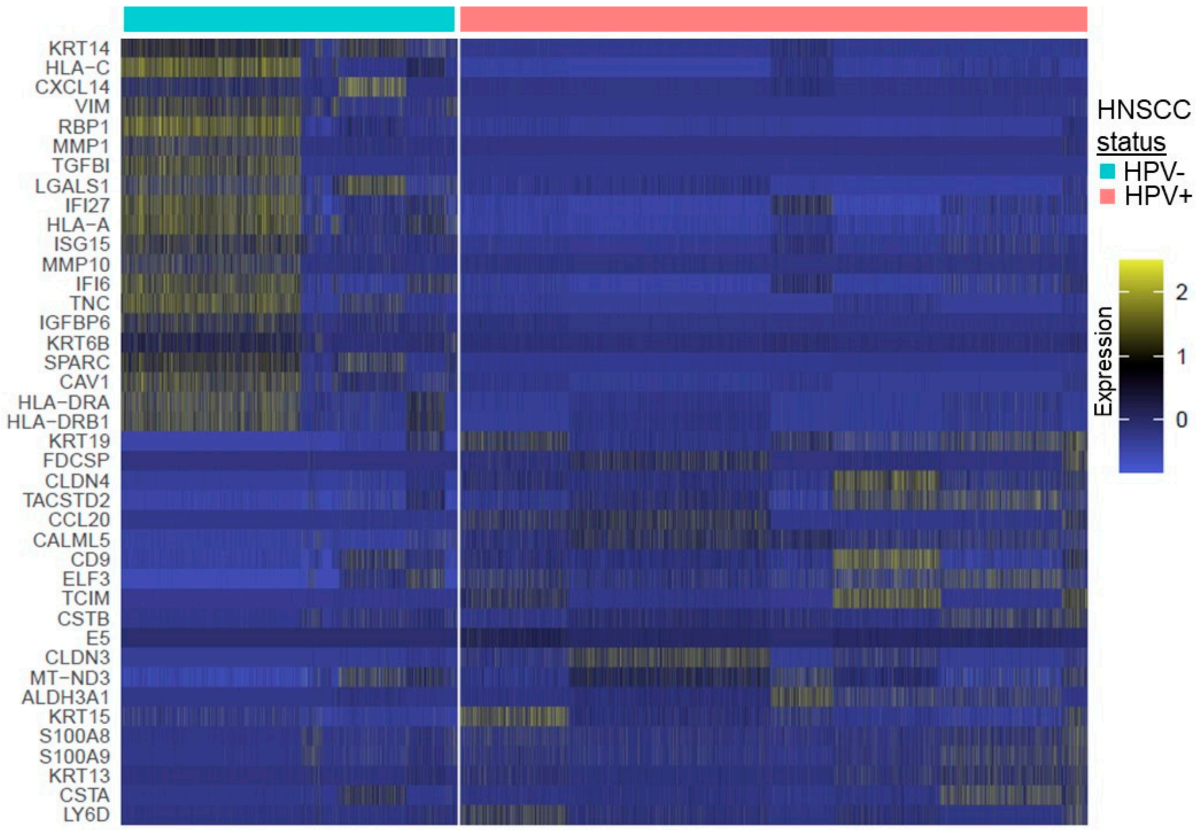

Supplementary Figure S1. Heatmap of genes differentially expressed in epithelial cells in HPV- vs HPV+ HNSCC tumors.

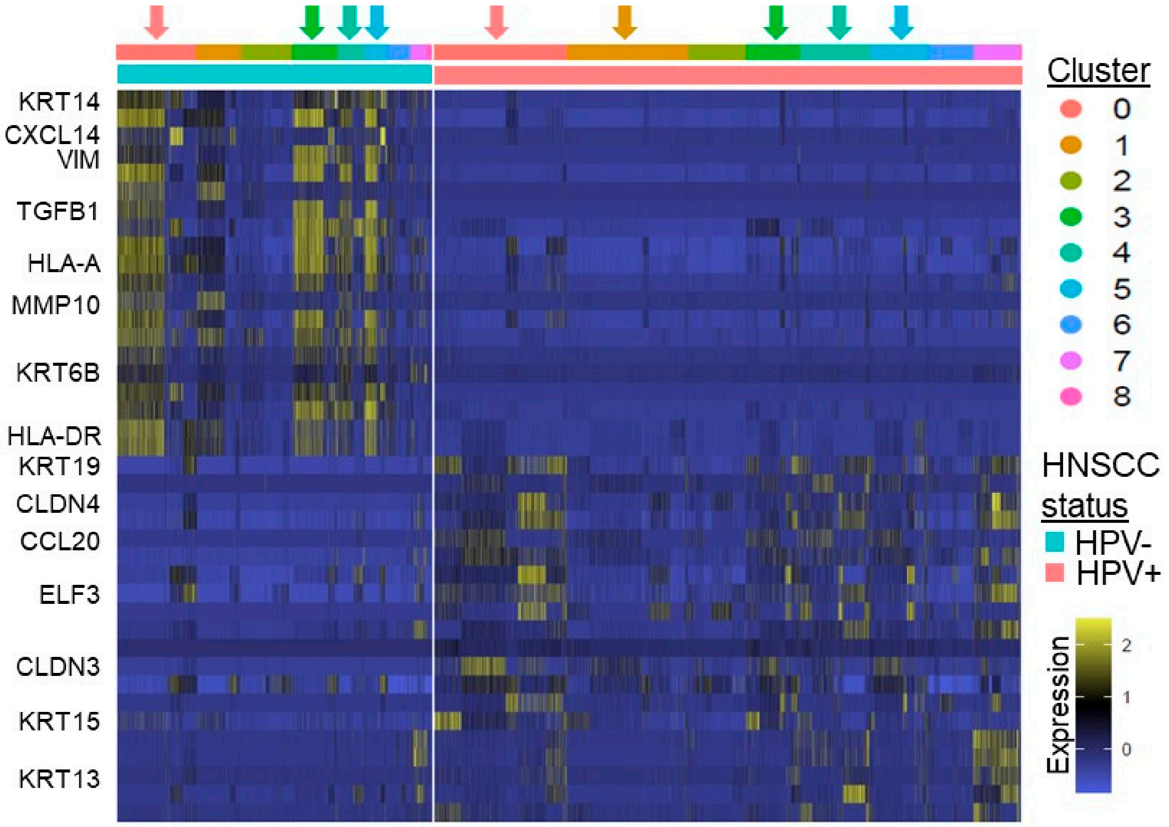

Supplementary Figure S2. Heatmap of genes differentially expressed in epithelial cells in HPV- vs HPV+ HNSCC tumors organized by clusters, with select clusters with distinct differences in gene profiles highlighted with arrows.
